# Radiomics Feature Activation Maps as a New Tool for Signature Interpretability

**DOI:** 10.3389/fonc.2020.578895

**Published:** 2020-12-08

**Authors:** Diem Vuong, Stephanie Tanadini-Lang, Ze Wu, Robert Marks, Jan Unkelbach, Sven Hillinger, Eric Innocents Eboulet, Sandra Thierstein, Solange Peters, Miklos Pless, Matthias Guckenberger, Marta Bogowicz

**Affiliations:** ^1^ Department of Radiation Oncology, University Hospital Zurich and University of Zurich, Zurich, Switzerland; ^2^ Department of Thoracic Surgery, University Hospital Zurich and University of Zurich, Zurich, Switzerland; ^3^ Department of Clinical Trial Management, Swiss Group for Clinical Cancer Research (SAKK) Coordinating Center, Bern, Switzerland; ^4^ Department of Oncology, Centre Hospitalier Universitaire Vaudois (CHUV), Lausanne, Switzerland; ^5^ Department of Medical Oncology, Kantonsspital Winterthur, Winterthur, Switzerland

**Keywords:** lung cancer, computed tomography, peritumoral radiomics, radiomics activation maps, local radiomics, interpretability

## Abstract

**Introduction:**

In the field of personalized medicine, radiomics has shown its potential to support treatment decisions. However, the limited feature interpretability hampers its introduction into the clinics. Here, we propose a new methodology to create radiomics feature activation maps, which allows to identify the spatial-anatomical locations responsible for signature activation based on local radiomics. The feasibility of this technique will be studied for histological subtype differentiation (adenocarcinoma *versus* squamous cell carcinoma) in non-small cell lung cancer (NSCLC) using computed tomography (CT) radiomics.

**Materials and Methods:**

Pre-treatment CT scans were collected from a multi-centric Swiss trial (training, n=73, IIIA/N2 NSCLC, SAKK 16/00) and an independent cohort (validation, n=32, IIIA/N2/IIIB NSCLC). Based on the gross tumor volume (GTV), four peritumoral region of interests (ROI) were defined: lung_exterior (expansion into the lung), iso_exterior (expansion into lung and soft tissue), gradient (GTV border region), GTV+Rim (GTV and iso_exterior). For each ROI, 154 radiomic features were extracted using an in-house developed software implementation (Z-Rad, Python v2.7.14). Features robust against delineation variability served as an input for a multivariate logistic regression analysis. Model performance was quantified using the area under the receiver operating characteristic curve (AUC) and verified using five-fold cross validation and internal validation. Local radiomic features were extracted from the GTV+Rim ROI using non-overlapping 3x3x3 voxel patches previously marked as GTV or rim. A binary activation map was created for each patient using the median global feature value from the training. The ratios of activated/non-activated patches of GTV and rim regions were compared between histological subtypes (Wilcoxon test).

**Results:**

Iso_exterior, gradient, GTV+Rim showed good performances for histological subtype prediction (AUC_training_=0.68–0.72 and AUC_validation_=0.73–0.74) whereas GTV and lung_exterior models failed validation. GTV+Rim model feature activation maps showed that local texture feature distribution differed significantly between histological subtypes in the rim (p=0.0481) but not in the GTV (p=0.461).

**Conclusion:**

In this exploratory study, radiomics-based prediction of NSCLC histological subtypes was predominantly based on the peritumoral region indicating that radiomics activation maps can be useful for tracing back the spatial location of regions responsible for signature activation.

## Introduction

Personalization of therapy options for patients with oncological diseases has gained great importance in recent years. Differentiation of non-small cell lung cancer (NSCLC) patients into histological subtypes, i.e., lung adenocarcinoma (ADC, ~50%) and squamous cell carcinoma (SCC, ~40%) (1) is for example an important factor in the choice of systemic treatments (2). Current biomarker assessments are often based on invasive interventions to extract a single-pin-pointed measurement. Consequently, there are many clinical scenarios with a clinical need for alternatives to tissue-based assessment of tumor histology: e.g., challenging anatomical locations for biopsy, unfavorable risk-benefit ratio for biopsy, history of more than one malignancy, or characterization of two simultaneously identified lung nodules.

Quantitative, image-based biomarkers, so-called radiomic features, can potentially overcome these obstacles (3–6). Extracted from medical images such as computed tomography (CT), those features rely on mathematical definitions to depict image-related characteristics. Features can often be subdivided into four main types: shape, intensity, texture and filtered based features, providing a 3D profile of the region of interest (ROI) (2). Radiomics has shown increasingly its potential usefulness in diagnosis, prognosis and response assessment (4, 6–8). For example, Aerts *et al*. showed that CT based radiomics was able to predict overall survival (OS) in NSCLC and head and neck cancer patients (concordance index=0.65, 0.69, respectively) treated with radiochemotherapy (5). Further, radiomics was reported prognostic for NSCLC patients treated with targeted therapies such as nivolumab, docetaxel and gefitinib with promising results (9, 10). Next to OS, other endpoints have been reported such as disease-free survival (11) or distant metastasis (12). Moreover, radiomics has shown to be useful for response assessment, i.e., in prediction of pathological complete response (13, 14). Identified radiomics features prognostic for survival in NSCLC were associated with image related tumor heterogeneity in CT imaging (15), i.e., entropy (16) or busyness (7) based on filtered images.

However, the quantitative and highly complex methodical nature of radiomics is a two-edged sword. Compared to manually assessed measures in radiological reports, these radiomic features lack in their interpretability, challenging the methodology to emerge from a research topic to a useful tool in clinical settings.

Gradually, this hurdle has been recognized and few research groups have attempted to improve the feature interpretability. One strong motion is to correlate radiomic features with known biological markers such as human papillomavirus (17, 18) or epidermal growth factor receptor (19–21). However, the biological data is often only of limited availability. In contrast, local radiomic features can be used to provide more spatial information about given signatures. Local radiomics refers to the extraction of radiomic features from small sub-regions (patches), which cover the complete ROI. Compared to traditional global radiomic features, the spatial location of these patches is known and hence differences in radiomics signatures can be determined on a smaller spatial scale. Bogowicz et al. for example showed that local radiomics differed substantially between recurrent to non-recurrent regions in head and neck cancer treated with radiotherapy (22). Local radiomics may not only serve as a detection tool, but the additional spatial information obtained from the patches potentially allows to trace the regions which are most revealing for a particular radiomics signature.

It is the aim of this exploratory study to create and analyze CT radiomics signature activation maps using local radiomics. As a case-study, we built tumoral and peritumoral radiomics models using a multi-centric imaging dataset to predict NSCLC histological subtypes. Local radiomic features were extracted for the model features to create radiomics feature activation maps. These maps were assessed to evaluate whether the tumoral or peritumoral region is more informative for NSCLC histology differentiation in pre-treatment CT.

## Materials and Methods

### Patient and Imaging Characteristics

Patient and imaging characteristics were integrated from a previous study (23). For the training cohort, pre-treatment CT scans were collected from 73 stage IIIA/N2 NSCLC patients from a prospective Swiss multi-centric randomized phase 3 trial (SAKK 16/00 (5), neoadjuvant chemotherapy or radiochemotherapy prior to surgery). For the validation cohort, CT scans of 32 stage IIIA/N2 or IIIB NSCLC patients were included (induction radiochemotherapy or chemotherapy only prior to surgery) which were treated at the University Hospital Zurich (USZ). Patients with histological subtypes ADC and SCC were selected for this study. Histology as well as patient staging [6^th^ edition of the tumor-node-metastasis (TNM) classification] were defined according to the SAKK 16/00 protocol (5). Patients were similarly distributed between ADC and SCC subtype in the training and validation cohort (61.6 and 56.3% of ADC patients in training and validation, respectively) (**Supplement A, Table 1**).

Patients received non-contrast enhanced, non-gated pre-treatment CT scans reconstructed with filtered-back projection (FBP) using standard convolution kernel. Due to the multi-centric imaging set, we defined the standard kernel as follows: GE—STANDARD, Siemens—B30f/B31f, Toshiba—FC18, and Philips—B, similarly to the phantom study of Mackin and Ger et al. *(* 24, 25). CT spatial resolution varied between 0.98 and 1.37 mm in-plane and 0.6 to 5.0 mm slice thickness. Patients from the validation cohort received a non-contrast enhanced average CT and were imaged on CT scanner Discovery RX, STE, 690 and Biograph 128 Edge, 128, 40, 6 and SOMATOM Definition AS, from GE MEDICAL SYSTEMS and SIEMENS. Scans were reconstructed with FBP and a smooth kernel (STANDARD, I30f, B31f). CT spatial resolution was 0.98, 1.17, 1.37 mm in-plane and 2 and 3.27 mm slice thickness.

### Delineation

**Figure 1:**
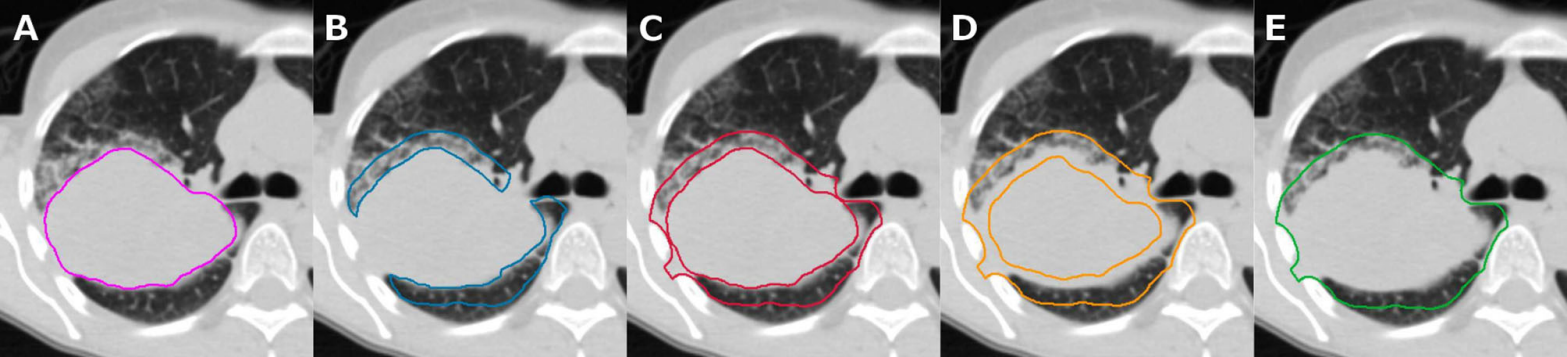
Same axial slice of a patient in our cohort shown for tumoral and peritumoral region of interests (ROIs), i.e., visual extent of the primary tumor (**A**, GTV), 0.8 cm expansion into lung tissue region inside the lung (**B**, lung_exterior), 0.8 cm expansion into lung and soft tissue (**C**, iso_exterior), 0.4 cm contraction and 0.8 cm expansion from the GTV (**D**, gradient), and primary tumor including iso_exterior (**E**, GTV+Rim).

Five ROIs were defined for this study (**Figure 1**):

- **GTV**: visual extent of the gross tumor volume (GTV)
- **lung_exterior**: 0.8 cm expansion from the GTV into lung tissue only
- **iso_exterior**: 0.8 cm expansion from the GTV into lung and soft tissue
- **gradient:** 0.4 cm contraction and 0.8 cm expansion from the GTV
- **GTV+Rim**: union of GTV and 0.8 cm expansion from GTV (iso_exterior)

The GTV ROI was manually delineated on the CT scans by an experienced physician using MIM VISTA (Version 6.7.9., MIM Software Inc., Cleveland, USA) with the lung window level and the support of registered PET images. All ROIs except for the GTV will be referred to as peritumoral ROIs. These peritumoral ROIs were created using an in-house developed MIM workflow. Anatomical structures which would strongly disturb the analysis, e.g., consisting of large air cavities (bronchi) or dense structures (bones) were manually excluded from all ROIs. Further, patients were excluded from the gradient analysis if the gradient regions comprised the entire GTV (**Figure 1D**).

### Robustness Study

The creation of the peritumoral ROIs was based on the manual delineation of the GTV, therefore a robustness study was performed to study the impact of inter-observer delineation variability on the radiomic features. A separate set of eleven patients were used as described in the study of Pavic et al. *(* 26). Three independent observers from USZ manually delineated the GTV. The same MIM workflow was used to create the peritumoral ROIs with the GTV of the three observers as an input. The intra-class correlation coefficient (ICC) was used as stability measure as described in Pavic et al. *(* 26). However, a stricter acceptance level of 0.9 was chosen, i.e., radiomic features with ICC > 0.9 were considered stable.

### Radiomics

Pre-treatment CT scans were resampled to 3.75 mm cubic voxels, the 75^th^ percentile of slice thicknesses in the training dataset using linear interpolation. Radiomic calculations were performed using an in-house developed software implementation (Z-Rad) based on Python programming language v 2.7.14 (for details on the software and features, please consult: https://medical-physics-usz.github.io). A Hounsfield unit (HU) range of −1,024 to 200 HU was chosen to exclude bone structures which could not be accounted for manually. Since the expansion and contraction parameters for the peritumoral ROIs were fixed, no shape features were considered for the analysis. Further, due to the small number of voxels in each direction, no wavelet features were included. Hence, a total of 154 radiomic features were calculated, i.e., intensity (n = 17) and texture (n = 137). Feature definitions were standardized according to the image biomarker standardization initiative (IBSI, version 11) (27). A fixed bin size of 20 HU was used to discretize the grey level values for texture analysis, resulting in approximately 60 bins, which has been shown to reduce intrinsic noise in the images while preserving essential texture (28).

### Statistical Analysis for Global Radiomics

To reduce the number of features, principal component analysis (PCA) was performed as a feature reduction method (29). The retained principal components were defined based on the 95% data variance. The feature which correlated the most with the selected principal component was used as a surrogate (the largest Pearson correlation coefficient). Univariate logistic regression analysis was performed to determine individual prognostic power of each features, separately. The significance level was 0.05, with no correction for multiple testing. Based on features with highest prognostic power per principal component group, a multivariate logistic regression model was built with backward selection using Akaike information criterion (AIC) which balances the goodness of fit of the model and its simplicity (30). The discriminatory power of the models was quantified using the area under the receiver operating characteristic curve (AUC) along with its 95% confidence interval (CI). Model performance was verified using 5-fold cross validation. Folds were chosen randomly without repetition. The generalizability of the models was verified in the validation cohort. Statistical analysis, model building and validation were performed with R [Version 3.5.1, packages: base, survival (31), survcomp (32), boot (33), pROC (34), and glmnet (35)].

### Creation of Activation Maps Based on Local Radiomics

**Figure 2:**
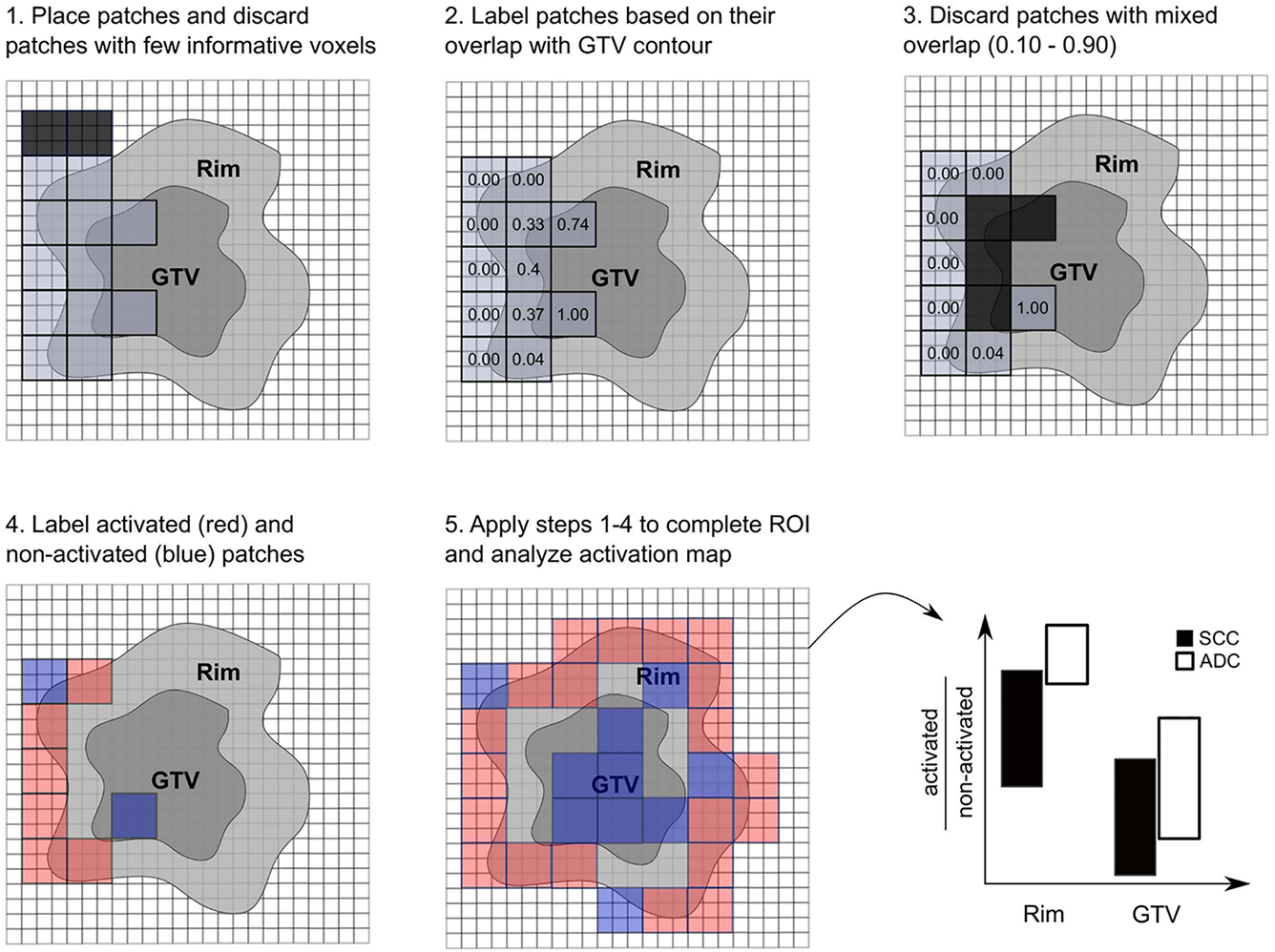
Scheme of radiomics feature activation map creation. Patches were optimally placed and patches with few informative voxels (< 9) were discarded (1), patches were labeled according to their overlap with the gross tumor volume (GTV) contour (2), and patches with mixed overlap were discarded (3). Patches were labeled activated (red) if their feature value was larger than the global median and were labeled non-activated (blue) if their feature value was smaller than the global median. The activation ratio was analyzed per region and histological subtype (5).

Local radiomic features were extracted from the GTV+Rim ROI using non-overlapping patches of size 3x3x3 voxels. This size of the patches allowed a meaningful calculation of the texture features (minimum number of voxels in each direction) as well as a meaningful overlap with the rim region (0.8 cm margin in each direction and 3.75 mm voxel size). The placement of the patches was automatically optimized to cover the entire ROI with a minimum number of patches. Patches with a low number of informative voxels (< 9 voxels) of the ROI were discarded. The overlap of the patches with the GTV was assessed, i.e., 100% referred to patches comprising only the GTV and 0% to patches comprising only normal tissue. This tool is intended to determine whether the radiomics signature for the prediction of histological subtypes originates from a certain predefined region. The signatures of patches with mixed overlap (10% to 90%) contain ambiguous information and were therefore discarded to clearly distinguish patches spatially assigned rim or GTV. Finally, patches with overlap lower than 10% or larger than 90% were labeled as rim and GTV, respectively. A binary feature activation map was created for each individual patient using the respective median of the global (standard) feature value in the training cohort, i.e., patches with feature value larger than the median were considered activated. The ratios of activated/non-activated patches for the normal tissue and the GTV were compared in the validation cohort between the histology types (Wilcoxon test), considering only patients with at least 27 patches and a minimum 3 patches per region (**Figure 2**, a more detailed description can be found in **Supplement D**).

## Results

### Modeling and Validation

Robust features were identified from the inter-observer variability robustness studies. Overall, the number of stable features for each ROIs were found to be moderate, i.e., GTV (49.7%), lung_exterior (57.6%), iso_exterior (57.6%), gradient (55.8%), and GTV+Rim (74.5%). The analysis can be found in the **Supplement B**. Results of the univariate analysis of the robust features selected in the feature selection step are shown in **Table 1**. Features marked with an asterisk were the final features retained after backward selection. Overall good univariate performances on the training set were observed (AUC = 0.61 to 0.72).

**Table 1:** Overview of univariate and multivariate analysis shown for all region of interests (ROIs) considered.

		Univariate		Multivariate	
ROI	Features	AUC	p-value	Coefficient	p-value
GTV	GLRLM_run entropy	0.64	0.034		
	GLCM_inverse variance*	0.65	0.035	−10.83	0.035
lung_exterior	GLSZM_zone percentage	0.63	0.046		
	GLCM_contrast	0.65	0.043		
	GLCM_homogeneity normalized*	0.72	0.004	52.51	0.004
	NGLDM_low dependence emphasis	0.66	0.025		
iso_exterior	Intensity_median	0.65	0.028		
	GLCM_correlation	0.61	0.043		
	GLSZM_zone size non-uniformity normalized*	0.68	0.015	−12.84	0.112
	Intensity_percentile_90*	0.68	0.025	−0.01	0.072
gradient	GLSZM_zone size non-uniformity normalized*	0.68	0.046	−20.26	0.046
GTV+Rim	Intensity_median*	0.63	0.026	0.002	0.144
	GLSZM_zone size non-uniformity normalized*	0.69	0.010	−16.75	0.026

Only features are listed which had a significant performance in the univariate analysis per principal component group. Features with an asterisk were retained in the final models after backward selection and their coefficients and p-values in the multivariate analysis are listed.

Different methods of feature selection were tested. The PCA + univariate logistic regression feature selection method led to simpler models. For majority of the ROIs, the models using PCA + univariate logistic regression performed best compared to other feature selection methods (**Supplement C**).

**Figure 3:**
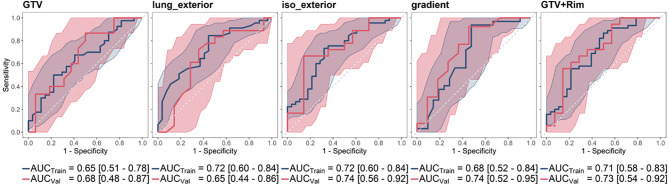
Receiver operating characteristic curve (ROC) curves and corresponding mean AUC [95% confidence interval] of the analyzed ROIs shown for training (blue) and validation cohort (red). The radiomic models based on GTV and lung_exterior could not be successfully validated in the validation cohort. Iso_exterior, gradient and GTV+Rim models had good performances in both cohorts.

For all regions, a logistic regression model could be built. The five-fold cross validation performance was [mean AUC (range)]: GTV [0.625 (0.23–1.00)], lung_exterior [0.72 (0.68–0.78)], iso_exterior [0.67 (0.46–0.84)], gradient [0.70 (0.48–0.82)], GTV+Rim [0.67 (0.48–0.84)]. The models based on the GTV and lung_exterior ROI were the only models which could not be validated on the validation cohort with 95% CI covering AUC = 0.5, i.e., a performance of a random predictor. Iso_exterior, gradient and the GTV+Rim showed acceptable performances in the range of 0.68–0.72 in the training and 0.73–0.74 in the validation cohort (**Figure 3**).

### Model Features

**Figure 4:**
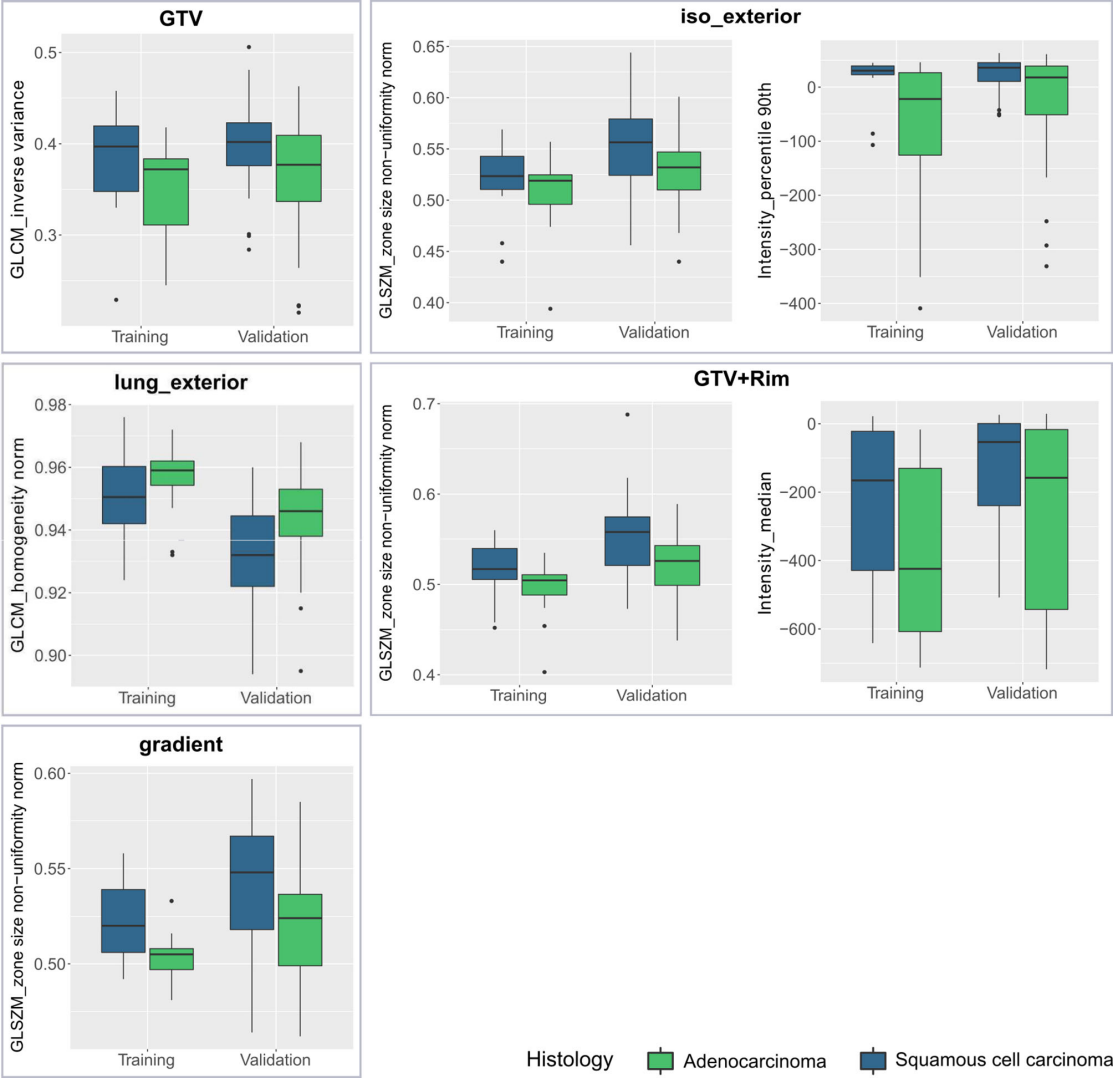
Boxplots of each studied region of interest (ROI) stratified by histological subtype (adenocarcinoma, squamous cell carcinoma) and dataset (training, validation) for the final model features.

In **Table 1**, the coefficients of the final model features are listed. The final models consisted of one or two features. Each model consisted of one texture features. These texture features can be associated with the texture heterogeneity in the ROI. For example, the gray level coocurrence matrix (GLCM) inverse variance in the GTV model is small if there is higher variance (**Figure 4**). The median GLCM inverse variance was lower for ADC compared to SCC, i.e., ADC were more likely to have heterogeneous and SCC more homogeneous patterns (**Figure 4**). For all regions, iso_exterior, gradient and the GTV+Rim, one texture feature (GLSZM_zone size non-uniformity normalized) was present in all three models. This feature counts the homogeneous zones of the same size over the different zone sizes and is low in patterns where zone counts are equally distributed along zone sizes, i.e., more heterogeneous patterns (**Figure 4**). In the models, the higher texture value (more homogeneous pattern) was associated more with SCC patients. Further, since this feature was present in the all three models, this feature will most likely be associated in the tumor adjacent region within the stable performing GTV+Rim model. Using the activation maps we further validate this assumption (see next section). Interestingly, the iso_exterior the 90% percentile intensity feature was significant more relevant in the model compared to the texture features whereas in the GTV+Rim model the opposite was observed (**Figure 4**).

### Analysis of Radiomics Feature Activation Maps

**Figure 5:**
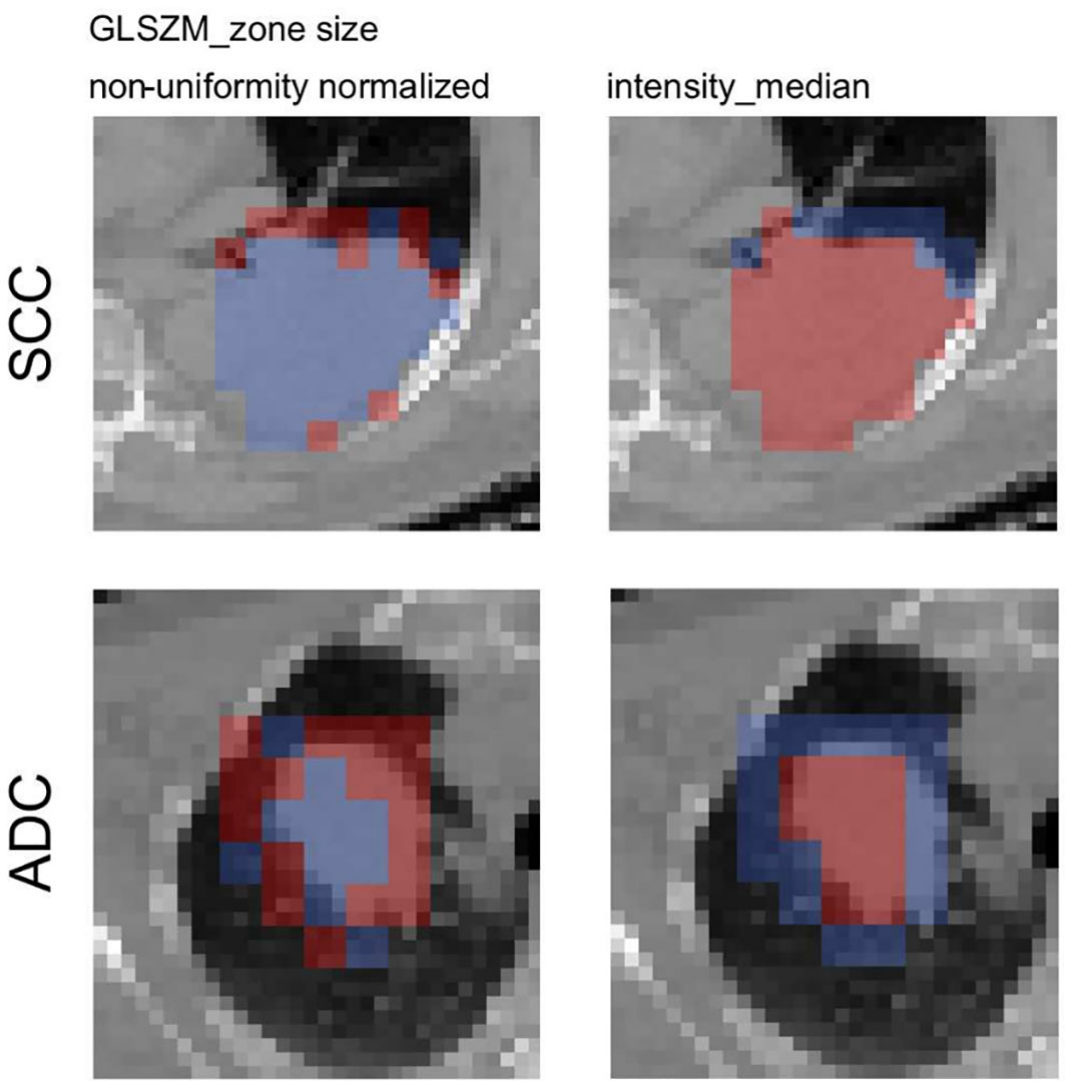
Axial slices of the feature activation maps overlaid with the corresponding CT scan of a squamous cell carcinoma patient (SCC) and an adenocarcioma patient (ADC) from the validation cohort. Activated (red) patches had feature values larger than the median feature value from the training, whereas non-activated (blue) patches had feature values smaller than the median. The texture feature was activated mostly in the rim region whereas the intensity_median was activated mostly in the tumor region.

The activation map analysis of the full radiomics signature indicated a greater importance of the rim region compared to the GTV (p=0.0541 and p=0.302 for rim and GTV, respectively). A closer analysis on the individual features showed that visually the texture feature was more activated on the adjacent region of the tumor, the intensity median more in the tumoral region. The median split values from the training cohort was 0.526 for GLSZM_zone size non-uniformity normalized and −158 HU for intensity_median. There was a significant difference in the activation ratio in the rim region when comparing ADC *vs.* SCC patients (p=0.048), however the ratio was non-significant in the tumor region (p=0.461). No significant difference in activated/non-activated ratio was observed in both regions for intensity median (**Figure 5**).

## Discussion

Ideally, clinically useful prognostic models should be performing reliably and be comprehensive. With the growing complexity of hand-crafted radiomic features, the feature interpretability becomes more relevant for its successful incorporation into clinical settings. Tools allowing feature interpretability may help in filtering false positive results in signature validation or clinical use.

In this exploratory study, we used a new local radiomics approach to create radiomics feature activation maps to locate the regions responsible for signature activation. On a local scale we were able to study whether the peritumoral or the tumoral radiomics was more informative for NSCLC histology differentiation in CT. To our knowledge, this is the first study to correlate peritumoral radiomic features with NSCLC histological subtypes. Multivariate logistic regression models were built for each ROI using features robust against inter-observer delineation variability. Iso_exterior, gradient as well as the combination of GTV and iso_exterior (GTV+Rim) showed acceptable performances in the range of AUC = 0.68–0.72 in the training and AUC=0.73–0.74 in the validation cohort whereas GTV and lung_exterior ROI models failed to validate. GTV+Rim radiomics feature activation maps for each patient showed that the rim region was more informative compared to tumoral radiomics to differentiate ADC and SCC.

CT based tumoral radiomic models have shown to be able to discriminate NSCLC histological subtypes, i.e., capturing that ADC cells are more loosely organized while SCC is more densely structured (36). Model performances however were not consistent across different studies ranging from moderate (37, 38) to good (36, 39–41). Possible explanation for worse performance of our GTV-based model compared to others may lay in the different imaging settings used. Reported models incorporated contrast-enhanced (17), respiratory-gated (15) CT scans or more complex modeling techniques such as Bayesian network (37). In our dataset, we selected a subset of patients with similar reconstruction settings resulting in small inter-scanner effects similar to previous studies (24, 25) potentially influencing the performance of the model. Further, scans were acquired in free-breathing which can introduce blurring to the final image (42, 43). However, in agreement with previous studies, ADC and SCC patients had a different tissue structure, i.e., the median of the mean intensity was significantly smaller for patients with ADC compared to SCC (p < 0.05). The final GTV model feature (GLCM_inverse variance) was lower for ADC patients compared to SCC patients, reflecting the more loosely structured tumor in ADC patients compared to more densely structured tissue of SCC patients. Lower GLCM_inverse variance feature can be associated with higher heterogeneity in the tissue in agreement with other studies (41), i.e., higher entropy values (associated with higher tumor heterogeneity) were observed to be associated with ADC tumors (40).

We hypothesized that peritumoral radiomics can depict better the known association between the anatomical tumor location and histological subtypes, i.e., ADC occur in more peripheral regions while SCC are often located centrally (1). This association is assumed to be most evident in iso_exterior ROI where the captured adjacent soft tissue structures can reflect the periphery or centrality of the primary tumor location. Indeed, the median 90% percentile in ADC was lower compared to SCC indicating less dense structures in the ROI. Further, it has been shown that the microscopic tumor extension in the peritumoral region differs between ADC and SCC. As a result, it has been suggested to use different margin sizes when treating ADC and SCC tumors to cover 95% of the microscopic tumor extension (8 and 6 mm margin for ADC and SCC, respectively) (44). With the chosen 8 mm margin for the iso_exterior region, this peritumoral model may depict this difference in the cell distribution.

The presence of the GLSZM_zone size non-uniformity normalized feature in the peritumoral ROI models indicated that this feature varies stronger between different histologies in the rim rather than in the GTV region in the GTV+Rim model. The activation maps of the GTV+Rim model confirmed this observation, i.e., the distribution of local texture feature of ADC differed significantly from SCC for the rim (p=0.048) but not for the GTV (p=0.461), irrespective that the feature threshold was based on the global feature values and no feature scaling was applied.

To account for the inter-observer delineation variability, a robustness study was performed for the primary tumor and peritumoral ROIs. The peritumoral feature stability was moderate, interestingly however, similar or even more stable than the primary tumor radiomics. A possible reason can be a different amount of lung tissue in the primary tumor delineation, which can result in higher sensitivity to manual delineations of the primary tumor compared to peritumoral regions where substantial lung tissue was *a priori* present. Further, an increased stability for larger ROI sizes can be observed. This observation is in agreement with Tunali et al.’s inter-observer variability study, where, however, the initial primary tumor contours were delineated using three semi-automatic segmentation methods (45). Arguably, the strict acceptance level ICC > 0.9, could have discarded potential useful features. However, due to the small cohort of 11 patients for the robustness analysis, the strict acceptance level helps ensuring that results were not affected by the small sample size. Recently, it has been shown that discarding features based on their robustness will lead to different models compared to modeling using a standardized imaging allowing to include all features (23).

The localization of signature relevant regions in the context of activation maps has been established in deep learning methods. Activation maps are pre-dominantly used to identify areas of interest used from the neural network to perform its class prediction. Introduction of such activation maps into the field of radiomics may provide an addition for clinical interpretability of radiomic models. In the context of peritumoral radiomics for example, where various peritumoral region definitions were reported in different sites (46), no clear strategy was available to determine the most promising region other than to model and validate each region individually. Therefore the tool presented in this study may guide the user to select the most relevant region in a more efficient way. Further, the presented tool can be not only applied on individual features but could be useful to interpret a complete signature for example by combining the activation maps of the model features. However, in our case, the texture feature had a more important role for the modeling compared to the intensity feature, therefore we did not include an analysis combining both activation maps.

It is important to study the link between local and global features. For intensity features, the global features do not necessary have to reflect the spatial saliency on a local scale, as they are not scale-invariant. For texture features, the distribution of discretized intensities need to be preserved between the local and global approach. For example, in our study, the same discretization for local and global radiomics was used (fixed bin size of 20 HU with bin 0 corresponding to minimum intensity in the entire ROI). For majority of texture features the link between global and local can be argued on the basis that they are calculated on the relationship of a single voxel to its immediate neighbor (e.g., GLCM, NGTDM) consistent with the definition of our patches (3x3x3 voxels). The patch size should be adjusted in situations where larger distances are used for the texture metrics calculation. In more complex metrics (e.g., GLSZM, GLRLM), further analysis is required to study the main driving factor of the feature values. However, in our study both iso_exterior and the GTV+Rim model shared the same feature (GLSZM_zone_size_non-uniformity_normalized) indicating a close link of that feature to the rim region, further also observed on the local scale. Similarly, another study showed that CT based local radiomics was useful to identifying subregions of head and neck tumors associated with different degrees of radiation curability, i.e., local features differed significantly between recurrent region and controlled (non-recurrent) region (22). In that study, heterogeneity on both global and local scale was linked to worse prognosis. Irrespectively, a closer investigation is needed to identify the optimal activation threshold.

This study has its limitations. Higher complexity features such as wavelet features were not included, since a minimum number of voxels in each direction is needed to provide a meaningful analysis. The published tumoral radiomic models consisted of filter-based features such as law-features or wavelet features. However, these features were also more sensitive to delineation variability (45). Further, strict cut-off values were chosen to differentiate patches originated from the GTV and rim (10% and 90%). These results will likely change when using different cut-off values. Out of the scope of this exploratory study was the use of different margin sizes for the definition of peritumoral ROI as well as the inclusion of clinical known prognostic factors which might have improved the presented model performances. A further limitation is our assumption that the tumor spreads isotropically radial from the primary tumor center of mass. However, we distinguished a tumor spread into the lung-only regions with an isotropic spread. Lastly, the small sample size could have impacted the results, further analysis incorporating more imaging data would be desired.

## Conclusion

In this exploratory study we have shown that feature activation maps using local radiomics proved to be useful for tracing back the spatial location of regions responsible for signature activation. Radiomics feature activation map analysis indicated that the rim region, which is anatomically the tumor invasion front, was more relevant for histological subtype prediction than the GTV in CT imaging.

## Data Availability Statement

The data analyzed in this study is subject to the following licenses/restrictions: radiomics data generated for this study are available on request from the corresponding author. Clinical data were provided by SAKK and are available upon request. Requests to access these datasets should be directed to DV, diem.vuong@usz.ch.

## Ethics Statement

The studies involving human participants were reviewed and approved by all involved Swiss cantonal ethics committees with the following reference numbers: EKNZ PB_2016-01071, KEK ZH PB_2016-00412, KEK Bern PB_2016-01072, CER-VD PB_2016-01078, CCER PB_2016-01073, EKOS PB_2016-01075, Comitato Etico Cantonale Bellinzona PB_2016-01077 for the training cohort as well as KEK ZH 2018-02405 for the validation cohort. The patients/participants provided their written informed consent to participate in this study.

## Author Contributions

Concept and design: DV, MB, ST-L. Acquisition and analysis: DV, MB, ZW, RM, JU. Data interpretation: DV, MB, ST-L, MG. Data collection: EE, ST, SH, MP, SP. Drafting of manuscript: DV, MB, ST-L. Critical revision of the manuscript: all authors. All authors contributed to the article and approved the submitted version.

## Funding

This work was supported by the Swiss National Science Foundation (310030_173303).

## Conflict of Interest

The authors declare that the research was conducted in the absence of any commercial or financial relationships that could be construed as a potential conflict of interest.

## References

[1] Chen Z Fillmore CM Hammerman PS Kim CF Wong K-K Non-small-cell lung cancers: a heterogeneous set of diseases. Nat Rev Cancer (2014) 14(8):535–46. 10.1038/nrc3775 PMC5712844 25056707

[2] Standfield L Weston AR Barraclough H Kooten MV Pavlakis N Histology as a treatment effect modifier in advanced non-small cell lung cancer: A systematic review of the evidence. Respirology (2011) 16(8):1210–20. 10.1111/j.1440-1843.2011.02025.x 21801275

[3] Lambin P Rios-Velazquez E Leijenaar R Carvalho S van Stiphout RGPM Granton P Radiomics: Extracting more information from medical images using advanced feature analysis. Eur J Cancer (2012) 48(4):441–6. 10.1016/j.ejca.2011.11.036 PMC4533986 22257792

[4] Lambin P Leijenaar RTH Deist TM Peerlings J de Jong EEC ven Timmeren J Radiomics: the bridge between medical imaging and personalized medicine. Nat Rev Clin Oncol (2017) 14(12):749–62. 10.1038/nrclinonc.2017.141 28975929

[5] Aerts HJ Velazquez ER Leijenaar RT Parmar C Grossmann P Carvalho S Decoding tumour phenotype by noninvasive imaging using a quantitative radiomics approach. Nat Commun (2014) 5:4006. 10.1038/ncomms5006 24892406 PMC4059926

[6] Gillies RJ Kinahan PE Hricak H Radiomics: Images Are More than Pictures, They Are Data. Radiology (2016) 278(2):563–77. 10.1148/radiol.2015151169 PMC4734157 26579733

[7] Bogowicz M Vuong D Huellner MW Pavic M Andratschke N Gabrys HS CT radiomics and PET radiomics: ready for clinical implementation? Q J Nucl Med Mol Imag. Off Publ Ital Assoc Nucl Med AIMN Int Assoc Radiopharmacol IAR Sect Soc Of (2019) 63(4):355–70. 10.23736/s1824-4785.19.03192-3 31527578

[8] Thawani R McLane M Beig N Ghose S Prasanna P Velcheti V Radiomics and radiogenomics in lung cancer: A review for the clinician. Lung Cancer (2018) 115:34–41. 10.1016/j.lungcan.2017.10.015 29290259

[9] Aerts HJWL Grossmann P Tan Y Oxnard GR Rizvi N Schwartz LH Defining a Radiomic Response Phenotype: A Pilot Study using targeted therapy in NSCLC. Sci Rep (2016) 6:33860. 10.1038/srep33860 27645803 PMC5028716

[10] Dercle L Fronheiser M Lu L Du S Hayes W Leung DK Identification of Non–Small Cell Lung Cancer Sensitive to Systemic Cancer Therapies Using Radiomics. Clin Cancer Res (2020) 26(9):2151–62. 10.1158/1078-0432.CCR-19-2942 PMC9239371 32198149

[11] Huang Y Liu Z He L Chen X Pan D Ma Z Radiomics Signature: A Potential Biomarker for the Prediction of Disease-Free Survival in Early-Stage (I or II) Non-Small Cell Lung Cancer. Radiology (2016) 281(3):947–57. 10.1148/radiol.2016152234 27347764

[12] Coroller TP Grossmann P Hou Y Rios Velazquez E Leijenaar RT Hermann G CT-based radiomic signature predicts distant metastasis in lung adenocarcinoma. Radiother Oncol (2015) 114(3):345–50. 10.1016/j.radonc.2015.02.015 PMC4400248 25746350

[13] Coroller TP Agrawal V Narayan V Hou Y Grossmann P Lee SW Radiomic phenotype features predict pathological response in non-small cell lung cancer. Radiother Oncol (2016) 119(3):480–6. 10.1016/j.radonc.2016.04.004 PMC4930885 27085484

[14] Coroller TP Agrawal V Huynh E Narayan V Lee SW Mak RH Radiomic-Based Pathological Response Prediction from Primary Tumors and Lymph Nodes in NSCLC. J Thorac Oncol (2017) 12(3):467–76. 10.1016/j.jtho.2016.11.2226 PMC5318226 27903462

[15] Ganeshan B Panayiotou E Burnand K Dizdarevic S Miles K Tumour heterogeneity in non-small cell lung carcinoma assessed by CT texture analysis: a potential marker of survival. Eur Radiol (2012) 22(4):796–802. 10.1007/s00330-011-2319-8 22086561

[16] Dercle L Ammari S Bateson M Durand PB Haspinger E Massard C Limits of radiomic-based entropy as a surrogate of tumor heterogeneity: ROI-area, acquisition protocol and tissue site exert substantial influence. Sci Rep (2017) 7(1):7952. 10.1038/s41598-017-08310-5 28801575 PMC5554130

[17] Leijenaar RT Bogowicz M Jochems A Hoebers FJ Wesseling FW Huang SH Development and validation of a radiomic signature to predict HPV (p16) status from standard CT imaging: a multicenter study. Br J Radiol (2018) 91(1086):20170498. 10.1259/bjr.20170498 29451412 PMC6223271

[18] Bogowicz M Riesterer O Ikenberg K Stieb S Moch H Studer G Computed Tomography Radiomics Predicts HPV Status and Local Tumor Control After Definitive Radiochemotherapy in Head and Neck Squamous Cell Carcinoma. Int J Radiat Oncol Biol Phys (2017) 99(4):921–8. 10.1016/j.ijrobp.2017.06.002 28807534

[19] Li S Ding C Zhang H Song J Wu L Radiomics for the prediction of EGFR mutation subtypes in non-small cell lung cancer. Med Phys (2019) 46(10):4545–52. 10.1002/mp.13747 31376283

[20] Rios Velazquez E Parmar C Liu Y Coroller TP Cruz G Stringfield O Somatic Mutations Drive Distinct Imaging Phenotypes in Lung Cancer. Cancer Res (2017) 77(14):3922–30. 10.1158/0008-5472.CAN-17-0122 PMC5528160 28566328

[21] Liu Y Kim J Balagurunathan Y Li Q Garcia AL Stringfield O Radiomic Features Are Associated With EGFR Mutation Status in Lung Adenocarcinomas. Clin Lung Cancer (2016) 17(5):441–8.e6. 10.1016/j.cllc.2016.02.001 27017476 PMC5548419

[22] Bogowicz M Pavic M Riesterer O Finazzi T Garcia Schüler H Holz-Sapra E CT radiomics differentiates levels of radiocurability in tumor subvolumes in head and neck cancer, ESTRO 2020 Abstract book. Available at: https://cld.bz/8huStZo/262/ (Accessed November 15, 2020).

[23] Vuong D Bogowicz M Denzler S Oliveira C Foerster R Amstutz F Comparison of robust to standardized CT radiomics models to predict overall survival for non-small cell lung cancer patients. Med Phys (2020) 47(9):4045–53. 10.1002/mp.14224 32395833

[24] Ger RB Zhou S Chi P-CM Lee HJ Layman RR Jones AK Comprehensive Investigation on Controlling for CT Imaging Variabilities in Radiomics Studies. Sci Rep (2018) 8(1):13047. 10.1038/s41598-018-31509-z 30158540 PMC6115360

[25] Mackin D Fave X Zhang L Yang J Jones AK Ng CS Harmonizing the pixel size in retrospective computed tomography radiomics studies. PLoS One (2017) 12(9):e0178524. 10.1371/journal.pone.0178524 28934225 PMC5608195

[26] Pavic M Bogowicz M Würms X Glatz S Finazzi T Riesterer O Influence of inter-observer delineation variability on radiomics stability in different tumor sites. Acta Oncol (2018) 57(8):1070–4. 10.1080/0284186X.2018.1445283 29513054

[27] Zwanenburg A Vallières M Abdalah MA Aerts HJWL Andrearczyk V Apte A The Image Biomarker Standardization Initiative: Standardized Quantitative Radiomics for High-Throughput Image-based Phenotyping. Radiol Publ Online March 10 (2020) 295(2):328–38. 10.1148/radiol.2020191145 PMC7193906 32154773

[28] Larue R van Timmeren JE de Jong EEC Feliciani G Leijenaar RTH Schreurs WMJ Influence of gray level discretization on radiomic feature stability for different CT scanners, tube currents and slice thicknesses: a comprehensive phantom study. Acta Oncol (2017) 56(11):1544–53. 10.1080/0284186x.2017.1351624 28885084

[29] Jolliffe IT Cadima J Principal component analysis: a review and recent developments. Philos Trans R Soc Math Phys Eng Sci (2016) 374(2065):20150202. 10.1098/rsta.2015.0202 PMC4792409 26953178

[30] Akaike H A new look at the statistical model identification. IEEE Trans Autom Control (1974) 19(6):716–23. 10.1109/TAC.1974.1100705

[31] Therneau TM Lumley T Package ‘survival’. R Top Doc (2015) 128:112.

[32] Canty A Ripley B boot: Bootstrap R (S-Plus) functions. R Package Version (2017) 1:3–20.

[33] Schröder MS Culhane AC Quackenbush J Haibe-Kains B survcomp: an R/Bioconductor package for performance assessment and comparison of survival models. Bioinformatics 2011 27(22):3206–8. 10.1093/bioinformatics/btr511 PMC3208391 21903630

[34] Robin X Turck N Hainard A Tiberti N Lisacek F Sanchez J-C pROC: an open-source package for R and S+ to analyze and compare ROC curves. BMC Bioinf (2011) 12(1):77. 10.1186/1471-2105-12-77 PMC3068975 21414208

[35] Friedman JH Hastie T Tibshirani R Regularization Paths for Generalized Linear Models via Coordinate Descent. J J Stat Softw (2010) 33(1):22. 10.18637/jss.v033.i01 2010. PMC2929880 20808728

[36] E L Lu L Li L Yang H Schwartz LH Zhao B Radiomics for Classifying Histological Subtypes of Lung Cancer Based on Multiphasic Contrast-Enhanced Computed Tomography. J Comput Assist Tomogr (2019) 43(2):300–6. 10.1097/RCT.0000000000000836 PMC6527094 30664116

[37] Wu W Parmar C Grossmann P Quackenbush J Lambin P Bussink J Exploratory Study to Identify Radiomics Classifiers for Lung Cancer Histology. Front Oncol (2016) 6:71. 10.3389/fonc.2016.00071 27064691 PMC4811956

[38] Parmar C Leijenaar RTH Grossmann P Radiomic feature clusters and Prognostic Signatures specific for Lung and Head & Neck cancer. Sci Rep (2015) 5(1):1–10. 10.1038/srep11044 PMC4937496 26251068

[39] Ferreira Junior JR Koenigkam-Santos M Cipriano FEG Fabro AT Azevedo-Marques PM de. Radiomics-based features for pattern recognition of lung cancer histopathology and metastases. Comput Methods Progr Biomed (2018) 159:23–30. 10.1016/j.cmpb.2018.02.015 29650315

[40] Digumarthy SR Padole AM Gullo RL Sequist LV Kalra MK Can CT radiomic analysis in NSCLC predict histology and EGFR mutation status? Med (Baltimore) (2019) 98(1):e13963. 10.1097/MD.0000000000013963 PMC6344142 30608433

[41] Zhu X Dong D Chen Z Fang M Zhang L Song J Radiomic signature as a diagnostic factor for histologic subtype classification of non-small cell lung cancer. Eur Radiol (2018) 28(7):2772–8. 10.1007/s00330-017-5221-1 29450713

[42] Oliver JA Budzevich M Zhang GG Dilling TJ Latifi K Moros EG Variability of Image Features Computed from Conventional and Respiratory-Gated PET/CT Images of Lung Cancer. Transl Oncol (2015) 8(6):524–34. 10.1016/j.tranon.2015.11.013 PMC4700295 26692535

[43] Lafata K Cai J Wang C Hong J Kelsey CR Yin F-F Spatial-temporal variability of radiomic features and its effect on the classification of lung cancer histology. Phys Med Biol (2018) 63(22):225003. 10.1088/1361-6560/aae56a 30272571

[44] Giraud P Antoine M Larrouy A Milleron B Callard P De Rycke Y Evaluation of microscopic tumor extension in non–small-cell lung cancer for three-dimensional conformal radiotherapy planning. Int J Radiat Oncol (2000) 48(4):1015–24. 10.1016/S0360-3016(00)00750-1 11072158

[45] Tunali I Hall LO Napel S Cherezov D Guvenis A Gillies RJ Stability and reproducibility of computed tomography radiomic features extracted from peritumoral regions of lung cancer lesions. Med Phys (2019) 46(11):5075–85. 10.1002/mp.13808 PMC6842054 31494946

[46] Keek S Sanduleanu S Wesseling F de Roest R van den Brekel M van der Heijden M Computed tomography-derived radiomic signature of head and neck squamous cell carcinoma (peri)tumoral tissue for the prediction of locoregional recurrence and distant metastasis after concurrent chemo-radiotherapy. PLoS One (2020) 15(5):e0232639. 10.1371/journal.pone.0232639 32442178 PMC7244120

