# Supplementary material for: Radiomics Feature Activation Maps as a New Tool for Signature Interpretability
Source: Front Oncol. 2020 Dec 8;10:578895. doi: 10.3389/fonc.2020.578895 (PMC7753181; doi:10.3389/fonc.2020.578895)
Supplement: Supplementary file 1 [file DataSheet_1.docx]

**Supplement A: Patient characteristics**

**Radiomics feature activation maps as a new tool for signature interpretability**

Vuong D^1^*, Tanadini-Lang S^1^, Wu Z^1^, Marks R^1^, Unkelbach J^1^, Hillinger S^2^, Eboulet E^3^, Thierstein S^3^, Peters S^4^, Pless M^5^, Guckenberger M^1^, Bogowicz M^1^

^1^ Department of Radiation Oncology, University Hospital Zurich and University of Zurich, Zurich, Switzerland

^2^ Department of Thoracic Surgery, University Hospital Zurich and University of Zurich, Zurich, Switzerland

^3^ Department of Clinical Trial Management, Swiss Group for Clinical Cancer Research (SAKK) Coordinating Center, Bern, Switzerland

^4^ Department of Oncology, Centre Hospitalier Universitaire Vaudois (CHUV), Lausanne, Switzerland

^5^ Department of Medical Oncology, Kantonsspital Winterthur, Winterthur, Switzerland

for the Swiss Group for Clinical Cancer Research (SAKK)

Table 1: Patient characteristics

|  | **Training (73)** | **Validation (32)** |
| --- | --- | --- |
| **Sex** |  |  |
| Female | 30 (41.10%) | 12 (37.50%) |
| Male | 43 (58.90%) | 20 (62.50%) |
| **Age** |  |  |
| Median | 60.00 | 69.09 |
| **Tumor Histology** |  |  |
| Adenocarcinoma | 45 (61.64%) | 18 (56.25%) |
| Squamous cell carcinoma | 28 (38.36%) | 14 (43.75%) |
| **Tumor Stage** |  |  |
| T1 | 11 (15.07%) | 1 (3.13%) |
| T2 | 42 (57.53%) | 12 (37.50%) |
| T3 | 18 (24.66%) | 10 (31.3%) |
| T4 | 2 (2.74%) | 9 (28.13%) |
| **Nodal Stage** |  |  |
| N0 | 0 (0.00%) | 2 (6.25%) |
| N1 | 0 (0.00%) | 4 (12.50%) |
| N2 | 73 (100.00%) | 26 (81.25%) |
| **Metastasis Stage** |  |  |
| M0 | 72 (98.63%) | 32 (100.00%) |
| M1 | 1 (1.37%) | 0 (0.00%) |
| **Treatment Arm** |  |  |
| Radiochemotherapy | 32 (43.84%) | 1 (3.13%) |
| Chemotherapy | 41 (56.16%) | 31 (96.87%) |
| **Survival** |  |  |
| OS @ 2 yrs | 35 (38.04%) | 7 (26.92%) |
| Median OS Time [Months] | 42.85 | 40.15 |


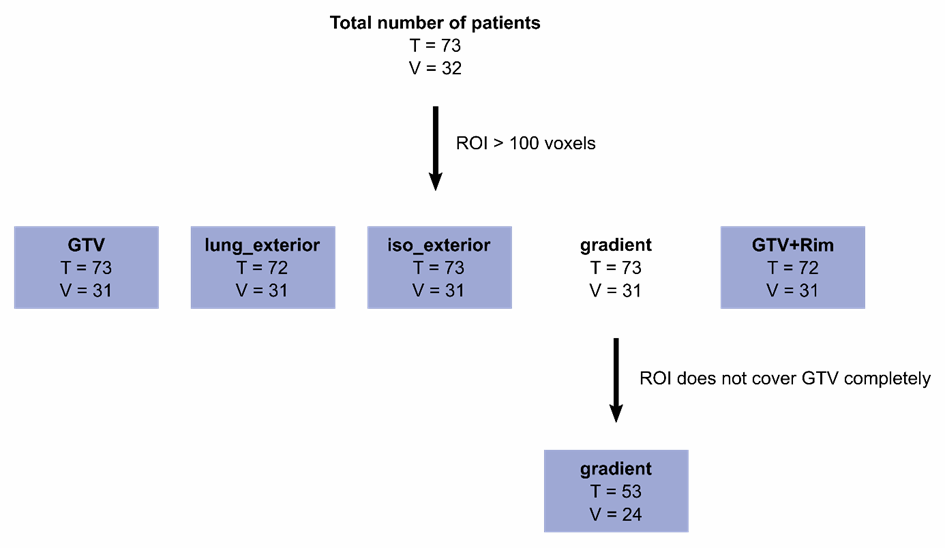
Figure 1: Scheme of patient selection for training (T) and validation (V) for each model. First, patients were excluded if the ROI contained less than 100 voxels. For the gradient ROI, additionally patient were excluded if the ROI enclosed the primary tumor completely.
