# Supplementary material for: Radiomics Feature Activation Maps as a New Tool for Signature Interpretability
Source: Front Oncol. 2020 Dec 8;10:578895. doi: 10.3389/fonc.2020.578895 (PMC7753181; doi:10.3389/fonc.2020.578895)
Supplement: Supplementary file 2 [file DataSheet_2.docx]

**Supplement B: Robustness studies**

**Radiomics feature activation maps as a new tool for signature interpretability**

Vuong D^1^*, Tanadini-Lang S^1^, Wu Z^1^, Marks R^1^, Unkelbach J^1^, Hillinger S^2^, Eboulet E^3^, Thierstein S^3^, Peters S^4^, Pless M^5^, Guckenberger M^1^, Bogowicz M^1^

^1^ Department of Radiation Oncology, University Hospital Zurich and University of Zurich, Zurich, Switzerland

^2^ Department of Thoracic Surgery, University Hospital Zurich and University of Zurich, Zurich, Switzerland

^3^ Department of Clinical Trial Management, Swiss Group for Clinical Cancer Research (SAKK) Coordinating Center, Bern, Switzerland

^4^ Department of Oncology, Centre Hospitalier Universitaire Vaudois (CHUV), Lausanne, Switzerland

^5^ Department of Medical Oncology, Kantonsspital Winterthur, Winterthur, Switzerland

for the Swiss Group for Clinical Cancer Research (SAKK)


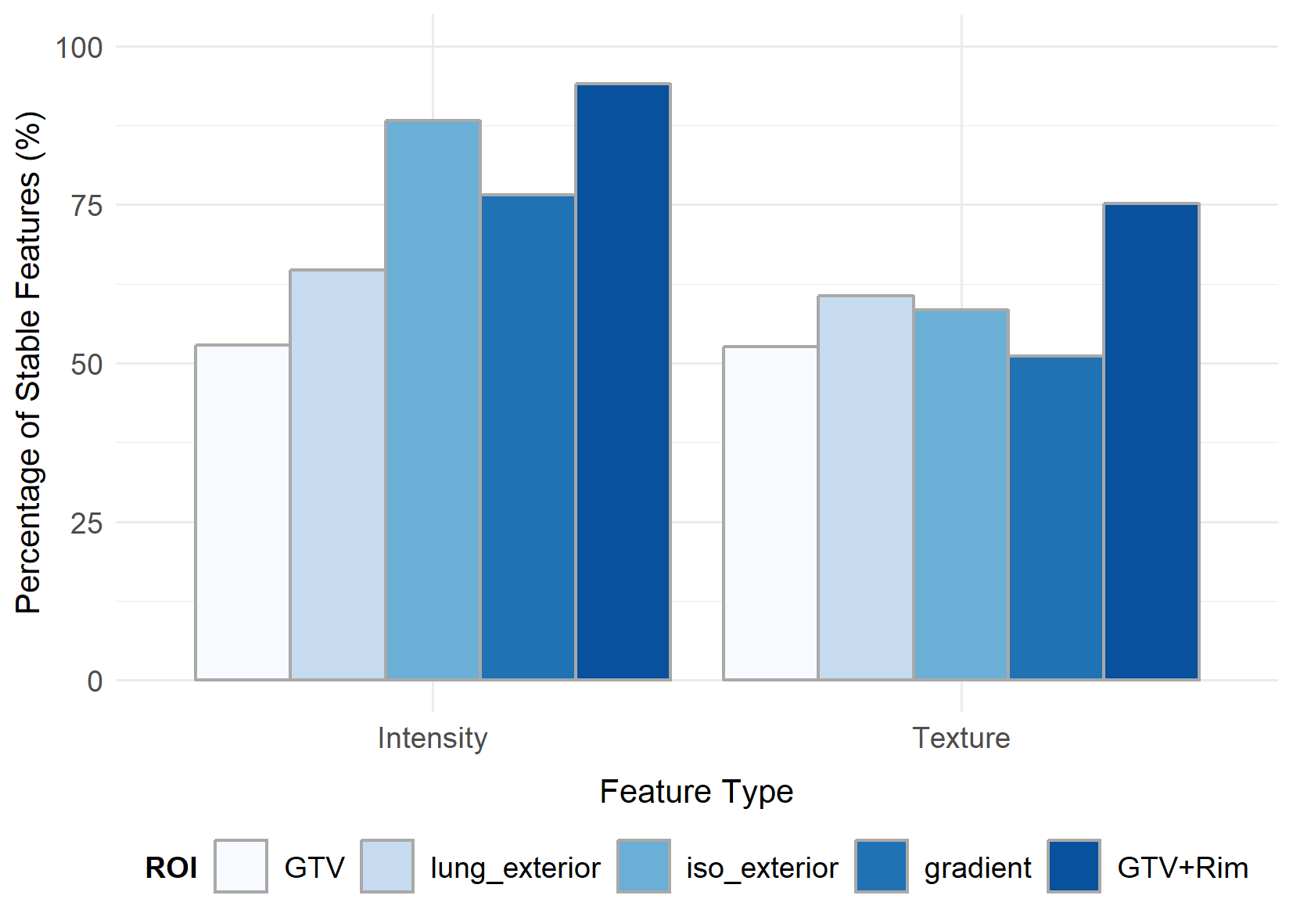


Figure 1: Robustness study on inter-observer delineation variability. Barplots of percentage of stable features for considered ROIs (GTV, lung_exterior, iso_exterior, gradient and GTV+Rim) stratified for intensity and texture features.

Initial inter-observer delineation variability of the primary tumor can influence the stability of radiomic features extracted from the peritumoral ROIs. Overall, the number of stable features for each ROIs were found to be moderate, i.e. GTV (49.7%), lung_exterior (57.6%), iso_exterior (57.6%), gradient (55.8%) and GTV+Rim (74.5%). Interestingly, the overall stability of the peritumoral features was larger compared to the GTV. This observation was evident in particular for intensity features, where the GTV+Rim ROI was the most stable (94.1%). In terms of texture features, the stability of the features was very similar among GTV, iso_exterior, lung_exterior and gradient. Similarly to the intensity features, texture features from GTV+Rim was the most stable ROI (75% of stable features) (Figure 1).
