# Supplementary material for: Radiomics Feature Activation Maps as a New Tool for Signature Interpretability
Source: Front Oncol. 2020 Dec 8;10:578895. doi: 10.3389/fonc.2020.578895 (PMC7753181; doi:10.3389/fonc.2020.578895)
Supplement: Supplementary file 3 [file DataSheet_3.docx]

**Supplement C: Comparison of feature selection methods**

**Radiomics feature activation maps as a new tool for signature interpretability**

Vuong D^1^*, Tanadini-Lang S^1^, Wu Z^1^, Marks R^1^, Unkelbach J^1^, Hillinger S^2^, Eboulet E^3^, Thierstein S^3^, Peters S^4^, Pless M^5^, Guckenberger M^1^, Bogowicz M^1^

^1^ Department of Radiation Oncology, University Hospital Zurich and University of Zurich, Zurich, Switzerland

^2^ Department of Thoracic Surgery, University Hospital Zurich and University of Zurich, Zurich, Switzerland

^3^ Department of Clinical Trial Management, Swiss Group for Clinical Cancer Research (SAKK) Coordinating Center, Bern, Switzerland

^4^ Department of Oncology, Centre Hospitalier Universitaire Vaudois (CHUV), Lausanne, Switzerland

^5^ Department of Medical Oncology, Kantonsspital Winterthur, Winterthur, Switzerland

for the Swiss Group for Clinical Cancer Research (SAKK)

This supplement summarizes different feature selection methods used in a sub-analysis. We have performed three different feature selection methods, i.e. PCA + univariate logistic regression, Least Absolute Shrinkage Selection Operator (LASSO) and Minimum Redundancy Maximum Relevance (mRmR), and two classifiers (multivariate logistic regression and LASSO). In mRmR, an ensemble approach was used.^1^ It was partiality combined with the principal component analysis. The feature count was defined as the number of principal components, which explains 95% of data variance. The mRmR was repeated 1000 randomly selecting samples using bootstrap procedure. The redundancy between the features was defined as the Pearson correlation. Features, which achieved at least 80% selection rate, were included in the final set. Table 1 lists the 5-fold cross validation results as well as the features in the final models. More details on the specific modeling technique were reported and illustrated in a previous article from our group.^2^

**References**

1. De Jay N, Papillon-Cavanagh S, Olsen C, El-Hachem N, Bontempi G, Haibe-Kains B. mRMRe: an R package for parallelized mRMR ensemble feature selection. *Bioinformatics*. 2013;29(18):2365-2368. doi:10.1093/bioinformatics/btt383

2. Bogowicz M, Riesterer O, Stark LS, et al. Comparison of PET and CT radiomics for prediction of local tumor control in head and neck squamous cell carcinoma. *Acta Oncologica*. 2017;56(11):1531-1536. doi:10.1080/0284186X.2017.1346382

Table 1: Model performance results of the peritumoral radiomics models to predict histological subtypes in NSCLC. Three feature selection methods together with two classifiers were used and final features selected are listed. The 5-fold cross validation results were quantified using the area under the roc curve (AUC) and listed are the mean and the range in squared brackets.

|  | **GTV** | **lung_exterior** | **iso_exterior** | **gradient** | **GTV+Rim** | |
| --- | --- | --- | --- | --- | --- | --- |
| *PCA + univariate logistic regression + multivariate logistic regression* | | | | | |  |
| *CV* | 0.62 [0.23-1.00] | 0.72 [0.69-0.78] | 0.67 [0.46-0.84] | 0.69 [0.48-0.82] | 0.67 [0.48-0.84] | |
| *Features* | GLCM inverse variance | GLCM_homogeneity_n | GLSZM_zone size non-uniformity_n  Intensity_percentile_90 | GLSZM_zone size non-uniformity_n | Intensity_median  GLSZM_zone size non-uniformity_n | |
| univariate logistic regression + LASSO | | | | | |  |
| *CV* | 0. 66 [0.23 – 1.00] | 0.64 [0.52-0.71] | 0.62 [0.44-0.78] | * | 0.67 [0.48-0.87] | |
| *Features* | GLCM inverse variance  GLRLM_run_entropy | GLCM_contrast GLSZM_zone_percentage | GLSZM_zone size non-uniformity_n  Intensity_percentile_90  Intensity_median  GLCM_correlation | * | Intensity_median  GLSZM_zone size non-uniformity_n | |
| mRmR + multivariate logistic regression 80% hit rate threshold | | | | | |  |
| *CV* | 0.64 [0.25-1.00] | 0.72 [0.69-0.78] | 0.67 [0.42-0.82] | * | 0.64 [0.48-0.74] | |
| *Features* | Intensity_COV  GLRLM_run_entropy Intensity_percentile_90 | GLCM_homogeneity_n | GLSZM_zone size non-uniformity_n | * | NGTDM_busyness GLCM_maximal_correlation_coefficient  GLSZM_small_zone_low_grey_level_emphasis  GLSZM_zone size non-uniformity_n | |
| mRmR + LASSO | | | | | |  |
| *CV* | 0.66 [0.38-0.98] | * | * | * | 0.45 [0.28-0.55] | |
| *Features* | Intensity_COV  GLRLM_run_entropy | * | * | * | LCM_maximal_correlation_coefficient  GLSZM_small_zone_low_grey_level_emphasis | |
