# Supplementary material for: Radiomics Feature Activation Maps as a New Tool for Signature Interpretability
Source: Front Oncol. 2020 Dec 8;10:578895. doi: 10.3389/fonc.2020.578895 (PMC7753181; doi:10.3389/fonc.2020.578895)

**Supplement D: Creation of radiomics activation maps**

**Radiomics feature activation maps as a new tool for signature interpretability**

Vuong D^1^*, Tanadini-Lang S^1^, Wu Z^1^, Marks R^1^, Unkelbach J^1^, Hillinger S^2^, Eboulet E^3^, Thierstein S^3^, Peters S^4^, Pless M^5^, Guckenberger M^1^, Bogowicz M^1^

^1^ Department of Radiation Oncology, University Hospital Zurich and University of Zurich, Zurich, Switzerland

^2^ Department of Thoracic Surgery, University Hospital Zurich and University of Zurich, Zurich, Switzerland

^3^ Department of Clinical Trial Management, Swiss Group for Clinical Cancer Research (SAKK) Coordinating Center, Bern, Switzerland

^4^ Department of Oncology, Centre Hospitalier Universitaire Vaudois (CHUV), Lausanne, Switzerland

^5^ Department of Medical Oncology, Kantonsspital Winterthur, Winterthur, Switzerland

for the Swiss Group for Clinical Cancer Research (SAKK)

In the following, we present the detailed pipeline workflow to create radiomics activation maps. There are two distinct parts: the first part involves the global, traditional radiomics and the second the new local radiomics approach.

First, the region of interest (ROI) was contoured (1). The ROI used for the radiomics activation map computation was a union of the GTV (contoured manually) and a rim (defined as a 0.8 cm isotropic expansion of the GTV), also referred to as GTV+Rim. From this ROI, radiomic features of type intensity and texture were extracted for each patient (2). Feature selection, modeling and validation was performed as described in the manuscript (3). From here on, we move to a local radiomics approach to create radiomics feature activation maps. The new local approach starts by optimizing the patch placement to minimize the total number of patches (in our study 3x3x3 voxel patches were used, 4). Next, patches with few informative voxels (n<3), i.e. no overlap with the ROI contour were discarded (5). To assign the patches to GTV or the rim region, patches were labeled based on their overlap with the GTV contour. A patch with 1.0 was completely within the GTV and a patch with 0.0 completely within the rim (6). It is clear that patches can have a mixed overlap of GTV and rim. In this case, we discard these patches with mixed overlap (between 10% – 90%) to ensure distinct differentiation of the activation signature originating from the GTV and the one from the rim (7). The selected radiomic feature is then calculated for each individual patch resulting in a parametric feature map (8). To create an activation map, each individual patch will be labeled as activated or non-activated based on its feature value and global threshold. The global threshold is defined the median of the global radiomic feature (extracted from entire GTV+Rim) over the training cohort. This is the step where radiomics on a global scale is linked with radiomics on a local scale (9). Lastly, the activation ratio (#activated / #non-activated patches) in the rim and in the GTV are compared between adenocarcinoma and squamous cell carcinoma using statistical testing (10).


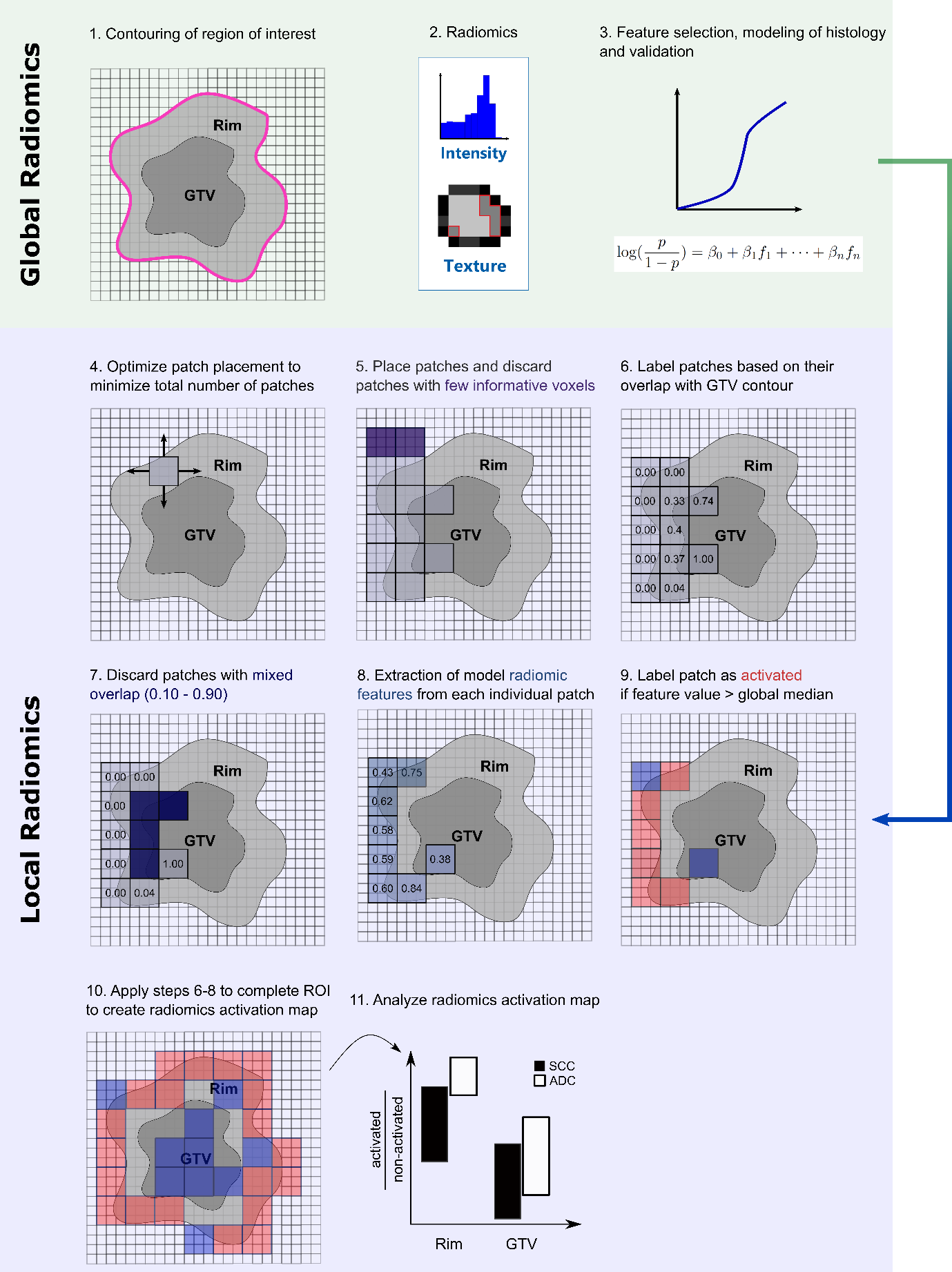

Supplement: Supplementary file 4 [file DataSheet_4.docx]
